# AMP-Activated Protein Kinase Signalling

**DOI:** 10.3390/ijms20030766

**Published:** 2019-02-12

**Authors:** Dietbert Neumann, Benoit Viollet

**Affiliations:** 1 Department of Pathology, CARIM School for Cardiovascular Diseases, Faculty of Health, Medicine and Life Sciences, Maastricht University, 6200 MD Maastricht, The Netherlands; 2 INSERM U1016, Institut Cochin, Department of Endocrinology, Metabolism and Diabetes (EMD), 24 rue du faubourg Saint-Jacques, 75014 Paris, France; 3 CNRS, UMR8104, 75014 Paris, France; 4 Université Paris Descartes, Sorbonne Paris Cité, 75014 Paris, France

AMP-activated protein kinase (AMPK) regulates energy homeostasis in eukaryotic cells and organisms. As such, AMPK has attracted enormous interest in various disciplines. Accordingly, the current Special Issue “AMP-Activated Protein Kinase Signalling” is a present-day reflection of the field covering a wide area of research. Although widely conserved throughout evolution and expressed ubiquitously, the functions of AMPK in different tissues and cell types may vary to some extent. Therefore, it is worthwhile to focus on cellular functions of various different origins, or tissues and organs, as well as their interplay in the context of the whole organism. This Special Issue includes research articles and reviews addressing AMPK regulation and function in all biological organization levels in health and disease.

Starting from the AMPK molecule, Yan et al. summarize the knowledge derived from crystal structures and provide expert insight into the molecular mechanisms of kinase activity modulation by adenine nucleotides [1]. As presented, recent research provided a detailed understanding of the molecular mechanisms leading to allosteric activation. The binding of AMP changes the AMPK’s conformational landscape, providing direct AMPK activation and protection against dephosphorylation of Thr-172 within the activation loop within the catalytic subunit. AMP bound at the cystathionine β-synthetase 3 (CBS3) nucleotide binding site within the regulatory AMPKγ subunit interacts with the flexible α-linker from the catalytic α subunit to transduce the adenine-binding signal to the kinase domain. The question arises as to how binding of AMP can inhibit dephosphorylation of Thr-172, while at the same time improving access to upstream kinases that phosphorylate the same site. Structural insight explaining the observed inhibition of AMPK by ATP is lacking at present and is identified as a key for understanding the regulation of AMPK activation loop phosphorylation. 

Apart from the allosteric mode of regulation, AMPK is part of a kinase cascade. Upstream regulation of AMPK involves one of several kinases capable of phosphorylating AMPK at Thr-172 in the α-subunit. Both liver kinase B1 (LKB1) and Ca^2+^/Calmodulin-dependent protein kinase kinase 2 (CaMKK2) are firmly established as physiological upstream kinases of AMPK. In addition, transforming growth factor β (TGF-β)-activated kinase 1 (TAK1) has also been reported as AMPK upstream kinase, but did not receive full attention as discussed in detail [2]. The historical origin of the conflict between researchers accepting TAK1 as a possible direct upstream kinase of AMPK and those rejecting this option is explained. Arguments from both sides lead to the conclusion that TAK1 should be accepted as a genuine contextual AMPK upstream kinase. Notably, the same contextual restriction applies to LKB1 and CaMKK2, which, depending on cell type, energy status, and environmental signal, act as alternative AMPK kinases. 

As reviewed by Janzen et al., in skeletal muscle, AMPK activity is regulated by glycogen content [3]. Glycogen physically binds AMPK, modifying its conformation to inhibit its activity. Vice versa, AMPK activity impacts glycogen storage dynamics to modulate exercise metabolism. In a monograph, Thomson summarizes AMPK signal integration in regulating skeletal muscle growth and atrophy [4]. Thomson suggests that activation of AMPKα1 mainly limits muscle growth, for example, by inhibiting protein synthesis, whereas AMPKα2 activation may play a more important role in muscle degradation, for example, through accelerating autophagy. Because a lack of AMPKα1 also inhibits muscle regeneration after injury, AMPKα1 may further have a mandatory function in regulating satellite cell dynamics. In general agreement, Vilchinskaya et al. describe AMPK as a key trigger in disuse-induced skeletal muscle remodelling [5]. In a mouse model overexpressing dominant negative AMPKα1 in skeletal muscle, Egawa et al. confirm the role of AMPK in muscle mass regulation upon unloading and reloading, but do not find evidence for AMPK involvement in fibre type switching [6]. The application of AMPK activating drugs to increase insulin sensitivity for improved glucose uptake in skeletal muscle is also a promising key therapeutic strategy to treat diabetes. Earlier results suggested the possible requirement of a serum factor in the insulin-sensitizing effect of the widely used AMPK activator 5-amino-imidazole-4-carboxamide ribonucleotide (AICAR). Jørgensen et al. clarify this issue by showing in mouse skeletal muscle that the beneficial effect of AICAR stimulation on downstream insulin signalling was not dependent on the presence of a serum factor [7].

Previous studies also reported that AMPK activation improved insulin sensitivity in endothelial cells for modulation of vascular homeostasis. Strembitska et al. investigate insulin-stimulated Akt phosphorylation in response to the AMPK activators AICAR, 991, and A-769662, the latter two chemicals targeting a different part of the AMPK molecule, the ADaM (allosteric drug and metabolism) site [8]. However, Strembitska et al., besides AMPK activation, observed AMPK-independent effects of A-769662 in human umbilical vein endothelial cells (HUVECs) and human aortic endothelial cells (HAECs) [8]. Namely, inhibition of insulin-stimulated Akt phosphorylation and nitric oxide (NO) synthesis by A-769662 was seen in the presence of AMPK inhibitor SBI-0206965. A-769662 also inhibited insulin-stimulated Erk1/2 phosphorylation in mouse embryo fibroblasts (MEFs) and in HAECs, which was independent of AMPK in MEFs, indicating that data obtained using this compound should be interpreted with caution [8]. Contradicting results have also been reported for AMPK-dependent regulation of endothelial NO synthase (eNOS). In endothelial cells, Zippel et al. observe AMPK-dependent inhibition of endothelial NO formation. The data provided suggest that AMPK targets Thr495 of eNOS, the inhibitory site, rather than Ser1177 (which would accelerate NO production) [9]. Notably, Zippel et al. applied genetic models of AMPK deficiency (CRISPR/Cas and mouse knockouts) and mutated eNOS at respective phosphorylation sites before incubating with AMPK in vitro, thus providing strong support for their physiological and mechanistic claims.

Hypertension and kidney disease can be a consequence of suboptimal early-life conditions, that is, by renal programming. Tain and Hsu bring forward the argument that AMPK activators could be applied for renal reprogramming as a protection against disease development [10]. Glosse and Föller review the involvement of AMPK in the regulation of renal transporters [11]. Without a particular focus on the kidney, but with relevance also for renal function, Rowart et al. describe the role of AMPK in the formation of epithelial tight junctions [12]. In particular, the authors discuss the contribution of AMPK in Ca^2+^-induced assembly of tight junctions.

Over the past decade, AMPK has emerged as a key player in the regulation of whole-body energy homeostasis. AMPK regulates food intake and integrates energy metabolism with several hormones, such as leptin, adiponectin, ghrelin, and insulin [13]. In this review, Wang et al. summarize the role of hypothalamic AMPK in hormonal regulation of energy balance. Nutrient intake, on the other hand, may regulate AMPK activation status. Lyons and Roche discuss the impact of dietary components on AMPK activity [14]. The authors review the evidence of whether specific nutrients and non-nutrient food components modulate AMPK-dependent processes relating to metabolism and inflammation, thus affecting the development of type 2 diabetes and obesity. Pointing out that the reported effects of diet on AMPK are mostly based on animal studies, the authors plead for further investigation in human studies. Resveratrol is one such nutritional substance that has been described as an AMPK activator. Trepiana et al. review the involvement of AMPK in the effects of resveratrol and its derivatives in the context of liver steatosis [15]. Although AMPK activation may only partly explain the preventive and therapeutic effects, the authors conclude that resveratrol represents a potential interesting approach to treat lipid accumulation in liver. Foretz et al. add further support for the potential of AMPK-dependent remodelling of lipid metabolism by providing in vivo evidence for increased fatty acid oxidation and reduced lipid content in mouse liver expressing constitutive active AMPK [16].

Apart from acute effects on the activity of enzymes or localization of proteins, AMPK has also been shown to change gene expression patterns for long-term adaptation involving regulation of transcription factors and chromatin remodelling. Gongol et al. describe AMPK as a key player in epigenetic regulation and discuss the consequent physiological and pathophysiological implications [17]. AMPK is involved in regulation of protein acetylation and itself receives regulation by acetylation, as reviewed by Vancura et al. [18]. Apart from epigenetic and transcriptional regulation, the acetylating and deacetylating events are linked to cellular metabolism, all of which in part is controlled by AMPK. A weighted gene co-expression network analysis was carried out to investigate the interaction of AMPK and autophagy gene products during adipocyte differentiation [19]. In fact, differentiation of cells by definition involves cellular remodelling and thus may generally require autophagy, which could be linked to AMPK. Indeed, AMPK has been recognized as a major driver of autophagy, as reviewed by Tamargo-Gómez and Mariño [20]. Jacquel et al. summarize the evidence that AMPK regulates myeloid differentiation [21]. Because autophagy appears to support myeloid differentiation, the authors suggest investigating the potential of AMPK activators as an anti-leukemic strategy.

Long-term memory depends on the induction of immediate early genes (IEGs). Didier et al. report that AMPK controls the expression of IEGs upon synaptic activation via the cAMP-dependent protein kinase (PKA)/cAMP response element binding (CREB) signalling pathway [22]. Although genetic evidence suggests the requirement of AMPK, the mechanism through which AMPK may regulate PKA activation remains elusive. The authors speculate that AMPK may be required to maintain ATP levels, as a requirement for formation of cyclic AMP. Thus, AMPK may play an indirect role in PKA activation upon synaptic activation.

While many studies focused their attention on the tumour-suppressor effect of AMPK activation, there is now growing evidence that AMPK plays a dual role in cancer, that is, inhibiting growth but enhancing survival. Adding to this discussion, Zhang et al. show that loss of AMPKα2 impairs sonic hedgehog medulloblastoma tumorigenesis [23]. Silwal et al. review the function of AMPK in host defence against infections [24]. As pointed out by the authors, AMPK also plays a dual role, suppressive or supportive for viral infections, depending on the type of virus. The role of AMPK in adaptive and innate immune response to infection of microbial and parasitic infections is also discussed.

Human reproduction represents a less mature field of AMPK research. Martin-Hidalgo et al. review the known cellular roles of AMPK in spermatozoa [25]. The argument is made that AMPK acts as key molecule linking the sperm’s energy metabolism and ability to fertilize. In the context of pregnancy complications in humans, Kumagai et al. discuss the possibility of further investigating AMPK activators as a treatment in a subset of conditions [26]. In their perspective, the authors discuss the possibility of AMPK regulation by catechol-*O*-methyltransferase (COMT). 

In summary, the current Special Issue provides a representative cross-section of AMPK research and topical reviews. Thanks to the authors submitting their precious work and insights that are presented in this Special Issue, our understanding of AMPK structure, function, and regulation has further progressed. Additionally, it turns out that AMPK biology is more complex than most of us originally anticipated, leading many of the contributing authors to highlight the fact that we still lack information and need to address new questions in subsequent studies. For example, the molecular structure of AMPK, although studied in great detail, does not provide information on the dynamic movements that are inherent to an allosteric enzyme. Moreover, “AMPK” is a heterogeneous mixture of twelve different heterotrimeric complexes (αβγ combinations of α1, α2, β1, β2, γ1, γ2, and γ3) without considering splice variants. The concept emerges that isoforms of AMPK localized at different subcellular compartments may respond to specific cues and regulate only a subset of cellular processes that are now collectively attributed to AMPK. Indeed, AMPK isoform selectivity to specific substrates may arise from a compartmentalized AMPK signalling, rather than from distinct intrinsic kinase substrate specificity. Hence, the spatiotemporal regulation of individual AMPK complexes in various tissues and metabolic conditions awaits further clarification. Furthermore, the development of AMPK activating drugs is constantly progressing behind the scenes, holding more promise than ever for the possible treatment of human disease. AMPK research does not stand still. The knowledge about AMPK accordingly will steadily increase. Besides, the variety of research topics relating to AMPK may continue to evolve. As we are already working on the next edition, we encourage the reader to consider submission of their upcoming AMPK-focused work to the successor Special Issue entitled “AMP-Activated Protein Kinase Signalling 2.0”. 
